# Letter to the editor regarding the paper “Evaluation of functional disability after Chikungunya infection”

**DOI:** 10.1590/0037-8682-0575-2019

**Published:** 2020-04-22

**Authors:** Marina Carvalho Arruda Barreto, Luciano Pamplona de Goes Cavalcanti, Shamyr Sulyvan de Castro

**Affiliations:** 1 Universidade Federal do Ceará, Fortaleza, CE, Brasil.

**Dear Editor:**

The study by Panato and collaborators entitled “Evaluation of functional disability after Chikungunya infection”^1^ is interesting because since the highlighted theme is also the subject of research in our projects. This study investigated whether patients in the chronic phase of chikungunya (CHIK) develop disability by estimating prevalence and assessing associated risk factors. The robust sample of 130 subjects and the prospect of working with disability are points to be commended. In addition, the provision of relevant information on sociodemographic characteristics, clinical profile, and therapeutic follow-up and data related to health service are other notable strengths of the study. However, we noted that the study uses the expression “functional disability” to express the disability study variable, which is not the variable measurement recommended by the World Health Organization (WHO)^2^. Furthermore, the study used the Roland-Morris (RM) questionnaire to assess disability. However, this questionnaire is used specifically to assess disability in patients with low back pain, a less prevalent arthralgia among people with CHIK^3^.

The WHO, recognizing the need for standardization of concepts, established the International Classification of Functioning, Disability and Health (ICF) in 2001. In this classification, which also presents a theoretical and conceptual framework for health, functioning is defined as the “umbrella term encompassing all body functions, activities and participation. It indicates the positive aspects of the interaction between an individual (with a health condition) and its context factors (personal and environmental factors)”, whereas disability serves as an umbrella term for impairments, activity limitations, or participation restriction^2^. By assessing activities of day living, pain, and function, as stated in the study^1^, the RM questionnaire does not assess the functioning or disability of a person in its entirety, once the structure, participation, and personal and environmental factors are not considered. Only impairments and activity limitations could be assessed using the selected questionnaire. The RM questionnaire was created in 1983 to assess disability^4^, which is before the release of the ICF. That is why the RM questionnaire does not bring in its core the functioning concept, as it is recommended by the WHO.

Another point that should be addressed here is the use of the RM questionnaire’s score as an expression of disability. The RM questionnaire was designed to assess disability according to the concepts before the ICF, specifically in persons with low back pain. Subsequently, in all questions, the low back pain is clearly stated^5^. Considering that the most frequent arthralgia among persons with CHIK occur in hands/wrists/metacarpals, ankles, and knees^3^ and not in the low back, the study could be underestimating the prevalence of disability in the sample population. As a significant scientific research on disability in persons with CHIK is unavailable, it is paramount that the scientific community works together to improve the quality of our research data. In this line, it should be a central issue a greater accuracy when assessing functioning/disability. 

We brought to light two important points that could be improved in future scientific investigations in the field of the health of people with CHIK: 1) the correct use of the term related to disability and 2) the necessity of more accurately assessing the prevalence of disability. To overcome this double barrier, we strongly suggest implementing the World Health Organization Disability Assessment Schedule - WHODAS 2.0. We believe that the use of an instrument that is not specific to a clinical complaint and is related to the ICF is more effective in reducing the risk of inconsistency in CHIK-related disability prevalence studies. This instrument was formulated by the WHO using the ICF conceptual framework and provides a standardized method for health and disability assessments. It is easy to access and apply and can analyze the construct as recommended. It aims to assess health and disability by providing total and domain values (cognition, mobility, self-care, getting along, life activities and participation). There are 3 versions, namely 36 questions, 12 questions, and 12 + 24^6^. The instrument was translated into Brazilian Portuguese, and the 36-question version was validated in Brazil for its use in people with CHIK^7^. We appreciate the opportunity to initiate the discussion presented in this study, and our main goal is to positively contribute to improvements in research on disability among people with CHIK.
